# Existe Relação entre Miocardite Aguda e a Permeabilidade Intestinal? Dois Biomarcadores nos Ajudam a Responder a esta Pergunta

**DOI:** 10.36660/abc.20230493

**Published:** 2023-08-28

**Authors:** Fernando Arturo Effio Solis, Adriana Brentegani, Marcelo Luiz Campos Vieira

**Affiliations:** 1 Hospital Israelita Albert Einstein São Paulo SP Brasil Hospital Israelita Albert Einstein, São Paulo, SP – Brasil; 2 Hospital Sírio-Libanês São Paulo SP Brasil Hospital Sírio-Libanês, São Paulo, SP – Brasil; 3 Universidade de São Paulo Faculdade de Medicina Hospital das Clínicas São Paulo SP Brasil Instituto do Coração do Hospital das Clínicas da Faculdade de Medicina da Universidade de São Paulo, São Paulo, SP – Brasil

**Keywords:** Miocardite, Biomarcadores, Permeabilidade Intestinal, Zonulina, Preserpsina

Miocardite é uma doença inflamatória cardíaca desencadeada pela ação de agentes infecciosos ou toxinas, que levam a lesão de miócitos por ação direta ou pela ativação exacerbada do sistema imune. Pode culminar em dilatação cardíaca, queda da fração de ejeção, arritmias ou até morte súbita.^1^ Chamamos de “miocardite aguda” o quadro clínico que se inicia há menos de 30 dias. De acordo com registros recentes, miocardite aguda é mais prevalente em adultos jovens, principalmente mulheres^2^ e sua causa é predominantemente infecciosa tendo enterovírus, coxsackie, parvovírus B19 e, mais recentemente, vírus da COVID-19 como exemplos de agentes etiológicos virais.^3,4^ Bactérias enteropatogênicas como Salmonella, Shigella e Campylobacter também são algumas das principais causas de miocardite identificadas em séries de casos.^5^ A zonulina é uma proteína que modula a permeabilidade intestinal. Sua expressão leva ao enfraquecimento das junções de oclusão presentes nas células epiteliais intestinais. Esta expressão é estimulada por certos enteropatógenos, facilitando a translocação de agentes e toxinas para dentro do organismo,^6,7^ levando a um estado de endotoxemia. A presepsina é tida como um marcador confiável de endotoxemia de baixo grau e, desta forma, um marcador indireto do aumento da permeabilidade intestinal.^8,9^

Neste estudo transversal observacional,^10^ os pesquisadores avaliaram a possível relação entre permeabilidade intestinal e miocardite aguda por meio da análise dos níveis séricos de zonulina e presepsina em grupo de pacientes com miocardite aguda em comparação com indivíduos sem a doença. A miocardite aguda foi definida a partir de história clínica e elevação laboratorial de biomarcadores como CK-MB e troponina-I. Foram observados 138 indivíduos consecutivos, 68 com miocardite aguda e 70 no grupo controle, não havendo diferença entre as características demográficas básicas entre os grupos. Os níveis de proteína C-reativa (PCR), fibrinogênio, pico de CK-MB e pico de troponina-I foram significativamente maiores no grupo de miocardite do que no grupo controle. É interessante citar também que o grupo de pacientes com miocardite apresentou um histórico significativamente maior de COVID-19 ou vacinação contra COVID-19 nos últimos seis meses em comparação ao grupo controle. Os pesquisadores descobriram que os níveis de zonulina e presepsina foram estatisticamente maiores no grupo de pacientes com miocardite (p<0,001). Inclusive, os pacientes que tinham níveis maiores de zonulina apresentaram menor fração de ejeção do ventrículo esquerdo, maiores índices de arritmia e maiores queixas gastrointestinais. Observou-se também que os níveis de zonulina estão positivamente relacionados com presepsina (r=0,461), pico de CK-MB (r=0,744) e pico de troponina (r=0,627), tais resultados mostram que os níveis de zonulina e presepsina estão associados de forma positiva à gravidade da lesão cardíaca, medida pelos marcadores CK MB e troponina. Em análise de regressão logística binária multivariada, tanto a presepsina quanto a zonulina foram identificadas como preditores independentes para a doença. Análise da curva ROC foi realizada evidenciando que os valores preditivos para miocardite aguda da zonulina e da presepsina foram estatisticamente significativos (p < 0,001, para ambas).^10^

Esses resultados sugerem que pode haver de fato, uma relação clara entre o aumento da permeabilidade intestinal, representado pelo aumento sérico de zonulina e presepsina, e o mecanismo fisiopatológico da miocardite aguda. Os pesquisadores sugerem que na vigência de uma infecção, o aumento da permeabilidade intestinal confirmado pelo aumento nos níveis de zonulina, levaria a uma exposição maior a uma série de patógenos, evidenciado pelo aumento da presepsina. Esta exposição elevada a tais patógenos desencadearia uma ativação secundária do sistema imunológico, mecanismo principal envolvido na inflamação miocárdica.

Como pontos positivos, ambos biomarcadores são não invasivos e fáceis de obter. Além disso, a sua capacidade de predizer a doença e de apontar para doença mais grave e para maior risco de complicação a partir de concentrações séricas mais elevadas ressalta como a dosagem de zonulina e presepsina pode ser promissora no diagnóstico e seguimento de pacientes com suspeita de miocardite. Além do mais, o seu uso em adição aos biomarcadores tradicionais seria uma opção mais econômica na prática clínica, uma vez que reduziria a aplicação de métodos diagnósticos invasivos, caros, e nem sempre disponíveis como biópsia endomiocárdica (BEM), ressonância magnética cardíaca (RMC) e coronariografia.

Apesar dos resultados e perspectivas apresentados, este estudo tem algumas limitações como o fato de ser um estudo com poucos pacientes e de centro único. Além disso, se limitou a uma dosagem única dos biomarcadores, deixando em aberto se a análise da curva da concentração sérica da zonulina e presepsina ao longo do tempo poderia ter maior utilidade no diagnóstico, seguimento e em predizer desfechos clínicos como cura ou complicações. Outra questão que enfraquece o estudo é a não correlação dos achados clínicos e laboratoriais com métodos diagnósticos de maior complexidade como BEM, RMC e até análise ecocardiográfica mais detalhada.

Em conclusão, o estudo apresenta uma nova visão no campo do que conhecemos sobre a miocardite, evidenciando uma possível relação direta com a permeabilidade intestinal, através das proteínas zonulina e presepsina. Essa descoberta pode revolucionar a forma como encaramos a doença na nossa prática clínica possibilitando novas condutas no diagnóstico, seguimento e tratamento da miocardite. Aprofundar-se no eixo intestino-coração pode abrir novos caminhos em uma face pouco conhecida da cardiologia. Desta forma, surge a expectativa de novos estudos que abordem o assunto para que, além de validar o que os pesquisadores nos mostraram, possamos ampliar cada vez mais o conhecimento nesta área.
