# Supplementary material for: Existe Relação entre Miocardite Aguda e a Permeabilidade Intestinal? Dois Biomarcadores nos Ajudam a Responder a esta Pergunta
Source: Arq Bras Cardiol. 2023 Aug 28;120(8):e20230493. [Article in Portuguese] doi: 10.36660/abc.20230493 (PMC10519227; doi:10.36660/abc.20230493)
Supplement: Translation

# Translation: Is There a Relationship Between Acute Myocarditis and Intestinal Permeability? Two Biomarkers Help Us Answer this Question

**Authors:** Fernando Arturo Effio Solis, Adriana Brentegani, Marcelo Luiz Campos Vieira

**Affiliations:** 1 São Paulo SP Brazil Hospital Israelita Albert Einstein, São Paulo, SP – Brazil; 2 São Paulo SP Brazil Hospital Sírio-Libanês, São Paulo, SP – Brazil; 3 São Paulo SP Brazil Instituto do Coração do Hospital das Clínicas da Faculdade de Medicina da Universidade de São Paulo, São Paulo, SP – Brazil

**Keywords:** Myocarditis, Biomarkers, Intestinal Permeability, Zonulin, Preserpsin

Myocarditis is an inflammatory heart disease triggered by the action of infectious agents or toxins, which lead to myocyte damage by direct action or by exacerbated activation of the immune system. It can culminate in cardiac dilation, drop in ejection fraction, arrhythmias, or even sudden death.^1^ We call “acute myocarditis,” the clinical condition that begins less than 30 days ago. According to recent records, acute myocarditis is more prevalent in young adults, mainly women,^2^ and its cause is predominantly infectious, with enterovirus, coxsackie virus, parvovirus B19, and, more recently, the COVID-19 virus as examples of viral etiological agentes.^3,4^ Enteropathogenic bacteria such as Salmonella, Shigella, and Campylobacter are also some of the main causes of myocarditis identified in case series.^5^ Zonulin is a protein that modulates intestinal permeability. Its expression leads to the weakening of occlusion junctions present in intestinal epithelial cells. This expression is stimulated by certain enteropathogens, facilitating the translocation of agents and toxins into the body,^6,7^ leading to a state of endotoxemia. Presepsin is considered a reliable marker of low-grade endotoxemia and an indirect marker of increased intestinal permeability.^8,9^

In this observational cross-sectional study,^10^ researchers evaluated the possible relationship between intestinal permeability and acute myocarditis by analyzing serum levels of zonulin and presepsin in patients with acute myocarditis compared to individuals without the disease. Acute myocarditis was defined based on clinical history and laboratory elevation of biomarkers such as CK-MB and troponin-I. A total of 138 consecutive individuals were observed, 68 with acute myocarditis and 70 in the control group, with no difference in basic demographic characteristics between groups. C-reactive protein (CRP) levels, fibrinogen, peak CK-MB and peak troponin-I were significantly higher in the myocarditis group than in the control group. It is also interesting to mention that the group of patients with myocarditis had a significantly higher history of COVID-19 or vaccination against COVID-19 in the last six months compared to the control group. The researchers found that zonulin and presepsin levels were statistically higher in the group of patients with myocarditis (p<0.001). Even patients with higher levels of zonulin had lower left ventricular ejection fraction, higher arrhythmia rates, and greater gastrointestinal complaints. It was also observed that zonulin levels are positively related to presepsin (r=0.461), CK-MB peak (r=0.744), and troponin peak (r=0.627); such results show that zonulin and presepsin levels are positively associated with the severity of cardiac injury, as measured by CK MB and troponin markers. In multivariate binary logistic regression analysis, presepsin and zonulin were identified as independent predictors of the disease. Analysis of the ROC curve was performed, showing that the predictive values for acute myocarditis of zonulin and presepsin were statistically significant (p < 0.001, for both).^10^

These results suggest that there may indeed be a clear relationship between the increase in intestinal permeability, represented by the increase in serum zonulin and presepsin, and the pathophysiological mechanism of acute myocarditis. The researchers suggest that in the presence of an infection, the increase in intestinal permeability, confirmed by the increase in zonulin levels, would lead to a greater exposure to a series of pathogens, evidenced by the increase in presepsin. This high exposure to such pathogens would trigger a secondary activation of the immune system, the main mechanism of myocardial inflammation.

On the plus side, both biomarkers are non-invasive and easy to obtain. In addition, their ability to predict disease and point to more severe disease and a greater risk of complications from higher serum concentrations underscores how zonulin and presepsin dosage can be promising in diagnosing and following up patients with suspected myocarditis. Furthermore, its use in addition to traditional biomarkers would be a more economical option in clinical practice since it would reduce the application of invasive diagnostic methods, which are expensive and not always available, such as endomyocardial biopsy (EMB), cardiac magnetic resonance (CMR) and coronary angiography.

Despite the results and perspectives presented, this study has limitations, such as being a single-center study with few patients. In addition, it was limited to a single measurement of biomarkers, leaving open whether the analysis of the curve of serum concentrations of zonulin and presepsin over time could be more useful in diagnosis, follow-up, and predicting clinical outcomes such as cures or complications. Another issue that weakens the study is the non-correlation of clinical and laboratory findings with more complex diagnostic methods such as EMB, CMR, and even more detailed echocardiographic analysis.

In conclusion, the study presents a new vision of what we know about myocarditis, showing a possible direct relationship with intestinal permeability through the proteins zonulin and presepsin. This discovery could revolutionize how we face the disease in our clinical practice, enabling new approaches to diagnosing, following up, and treating myocarditis. Going deeper into the gut-heart axis could open new paths in a little-known facet of cardiology. In this way, the expectation of new studies that address the subject arises so that, in addition to validating what the researchers have shown us, we can increasingly expand the knowledge in this area.
